# Statistical screening analysis of the chemical composition and kinetic study of phenol-formaldehyde resins synthesized in the presence of polyamines as co-catalysts

**DOI:** 10.1371/journal.pone.0195069

**Published:** 2018-05-31

**Authors:** Magdalena Cygan, Mariusz Szemień, Stanisław Krompiec

**Affiliations:** 1 Pfleiderer Silekol Sp. z o.o., Kędzierzyn-Koźle, Poland; 2 Institute of Chemistry, University of Silesia, Katowice, Poland; Ludwig-Maximilians-Universitat Munchen, GERMANY

## Abstract

The physico-chemical and application properties of phenol-formaldehyde resins used in the production of laminated plastics depend on such factors as: type and amount of catalyst, formaldehyde-to-phenol mole ratio, temperature and time of the synthesis process. The special impact on the reaction mechanism and kinetics, e.g. on the formation of mono-, di- and trihydroxymethyl derivatives of phenol (PhOH) is a consequence of the type and amount of the catalyst. This paper presents the results of optimisation research of resol resin synthesis catalysed by trimethylamine (TEA) carried out according to 3^2^ experimental design. The aim of the research was to determine the synthesis conditions under which it is possible to achieve products with reduced content of unconverted formaldehyde (CH_2_O), phenol and its hydroxymetyl derivatives, while maintaining the required physico-chemical properties. The process employing selected polyamines as well as TEA together with polyamine co-catalysts i.e. diethylenetriamine (DETA) and triethylenetetraamine (TETA) was also studied. The results were compared with those of the resins which were synthesised with the use of classic catalysts–ammonia (NH_3_) and triethylamine for which the content of CH_2_O, PhOH and its hydroxymethyl derivatives was respectively: NH3—5.13% and 46.27%, TEA—0.33% and 52.41%. In the case DETA was added, the content of phenol and its hydroxymethyl derivatives could be reduced by 52.49% in relation to the resin obtained with the use of TEA and by 46.19% in relation to the resin obtained with the use of ammonia. The formaldehyde content was reduced by 78.79% and 98.64%, respectively. When TETA was added as a co-catalyst, the content of phenol and its derivatives was reduced by 48.04% versus triethylamine-catalysed resin and by 41.15% versus ammonia-catalysed material. The reduction in formaldehyde content reached 84.85% and 99.03%, respectively. The results of kinetic study were also presented, the prediction accuracy of the proposed kinetic model is more than 98% for all the catalysts in the state variable space. Polyamine co-catalysts gave much higher rate constants (0.50 and 0.45 for TETA and DETA, respectively).

## Introduction

The resol resins are produced in the phenol-formaldehyde condensation process, in the presence of basic catalysts, under various temperature and time regimes. Although they were synthesised for the first time over 100 years ago, the present-day products of that type are still considered an important sector in the polymer chemistry. The physico-chemical properties of those resins are controlled by such factors as: type and amount of catalyst, formaldehyde-to-phenol mole ratio, process temperature and time. The type and amount of catalyst are decisive for the reaction mechanism and kinetics, e.g. for the formation of objectionable components, like: mono-, di- and trihydroxymethylphenols, and for the contents of unreacted formaldehyde and phenol [1–6].

The resol resins are predominantly employed in the production of laminated materials which are then used i.a. in the furniture sector, in the building industry, in transport and for the manufacture of electric insulation elements. The production process of those plastics is based on applying a resin to a substrate material, i.e. usually paper or textiles, and then on drying that composite in drying chambers [7–10]. Unwanted emissions and deposition of some resin components and/or substrate particulates in drying chambers and in ventilation ducts also take place during that process. That is disadvantageous not only from the viewpoint of resin losses and the need to clean up the production plant frequently; some harmful substances contained in resins may be released to the workplace atmosphere as well. The chromatographic analysis of deposit samples from ventilation systems and drying chambers revealed the presence of phenol and considerable amounts of its hydroxymethyl derivatives. The high-temperature process can also discharge unreacted formaldehyde which, pursuant to the new EU Directive, was classified as carcinogenic (cat. 1B) and mutagenic (cat. 2) from 2016.

Hence, there is a need to reduce the content of unconverted formaldehyde, phenol and its hydroxymethyl derivatives in the resin material.

The authors analysed the composition of commercial resins produced with the use of classic catalysts, e.g. NaOH or ammonia, and which are then employed in the manufacture of laminated plastics [11]. The analyses revealed the total content of phenol and its hydroxymethyl derivatives as high as 50% and the content of unreacted formaldehyde over 0.5%. Urea or other admixtures are added to such resins to reduce the content of unreacted formaldehyde to the required level <0.5%. Urea, however, causes higher nitrogen content, which is objectionable, and it impairs the application performance of the resins.

The regular resin which is used for the production of laminated plastics offers the physico-chemical properties as follows:

- Phenol content      - < 12.0%
- Formaldehyde content   - ≤ 0.5% (≤ 0.1% from 2016)
- Viscosity_(20°C)_      - < 500 mPas
- Gelation time_(150°C, 0.5 ml)_  - (130–220) s
- Non-volatile matter_(135°C)_  - (58–65)%
- Nitrogen content     - < 0.5%

Having in mind the above requirements, the authors carried out a research programme intended to reduce the content of harmful substances: phenol, hydroxymethylphenols and formaldehyde, in resins. Earlier research on the effects of the resin synthesis parameters, i.e.: mole ratio of reacting substances, reaction temperature, and first of all the type and amount of the catalyst, or catalytic systems [12] has proved that the application of the ammonia/diethylenetriamine catalytic system significantly reduces the content of phenol and hydroxymethylphenols. However, that catalytic system is highly active and substantially shortens the synthesis time, which increases the concentration of unreacted formaldehyde above the level of 1%.

A number of experiments using other amine catalysts were carried out within our research programme. The formaldehyde content was significantly reduced, i.e. below 0.5%, only when triethylamine was employed. However, the content of phenol and its hydroxymethyl derivatives remained too high at the same time.

Focusing our efforts predominantly on low contents of unconverted formaldehyde, we selected triethylamine as a reference material and as a basic component of a future catalytic system. The optimisation studies were carried out with the use of that catalyst, according to the mathematical design of experiments (3^2^), in order to define the effects of the catalyst amount and synthesis temperature on the compositions of the resin products, and in particular on the contents of unconverted formaldehyde, phenol and its hydroxymethyl derivatives. Physico-chemical properties of resins were analysed as well. The tests enabled to define the effects of the process parameters and to select the advisable synthesis conditions [13,14]. Those conditions were then adhered to in a series of syntheses in which selected polyamine catalysts, i.e. diethylenetriamine (DETA) and triethylenetetramine (TETA), were used. Some experiments also involved triethylamine and above mentioned amines as co-catalysts. The obtained resins were analysed for their compositions and physico-chemical properties. One should add that using the polyamine catalysts is unprecedented and is considered a crucial element of novelty in this work.

## Materials and methods

### Experimental

The following raw materials were employed in our experiments:

1. Phenol, analytically pure, from Sigma-Aldrich.
2. Formalin, stabilised with methanol, from LERG S.A.
3. Triethylamine, analytically pure, from Sigma-Aldrich.
4. Diethylenetriamine 99%, from Sigma-Aldrich.
5. Triethylenetetramine 99%, from Sigma-Aldrich.
6. 2-Hydroxymethylphenol 98%, from Sigma-Aldrich.
7. 4-Hydroxymethylphenol 98%, from Sigma-Aldrich.
8. 4-Ethylresorcinol–internal standard, from Sigma-Aldrich.

In order to reduce the volatile matter content in the phenol-formaldehyde resin intended for the production of laminated plastics, a series of syntheses was conducted, catalysed with triethylamine, according to a 3-level 2-factor (3^2^) experimental design. The following process parameters were selected for optimisation: temperature—T and amount of catalyst—n_cat_. Based on optimisation studies, conditions could be selected for the resin synthesis tests with the use of polyamines as catalysts, and with the use of triethylamine as a catalyst and polyamines as co-catalysts.

The resins were synthesised in a computer-controlled set composed of: 1 dm^3^ reactor placed on a load cell; heated vessels for raw materials, i.e. phenol and formalin; system for automatic supply of liquid and solid feeds, and temperature control system. The amounts of phenol and formaldehyde (47% aqueous solution) as charged to the reactor resulted from the assumed mole ratio (1: 1.15), while the amount of the catalyst resulted from the experimental design. After charging the feeds to the reactor, the synthesis was continued for 5 hours at the temperature which was in line with the experimental schedule. When the condensation reaction was complete, the reaction mixture was cooled down to 50°C and its concentration was increased in a laboratory vacuum evaporator to reach the water content of 6–7%. Then, methanol was added to the concentrated product at a constant amount of 20%. The product resins were analysed for their compositions and physico-chemical properties, like viscosity, non-volatile matter, gelation time, etc.

The kinetic studies were carried out according to the above mentioned procedure with the following difference: the reaction mixture was sampled at specific time intervals, each sample was immediately placed in the freezing mixture, at approx. -30°C, thereby the chemical reactions were effectively slowed down, after that the concentration of phenol and formaldehyde was determined by HPLC.

### Analytical methods

The following analytical methods were used during our research:

- high performance liquid chromatography, with mass spectroscopy detection (HPLC/MS), to identify phenol and its hydroxymethyl derivatives,
- high performance liquid chromatography, with DAD UV-VIS detection, for quantitative determination of formaldehyde as well as phenol and its hydroxymethyl derivatives.

The HPLC/MS analyses and quantitative HPLC determinations were conducted with the use of a Dionex Ultimate 3000 chromatograph equipped with a Hypersil Gold RP-18 column (250×4.6 mm). The following conditions were adopted both for phenol identification tests and for quantitative determination of phenol and its derivatives:

*Eluent*            - *water*, *methanol (gradient)*
*Flow rate*          - *1*.*0 ml/min*
*Measurement time*        - *60 min*
*Wavelength for UV/VIS detector* - *280 nm*

**Fig 1:**
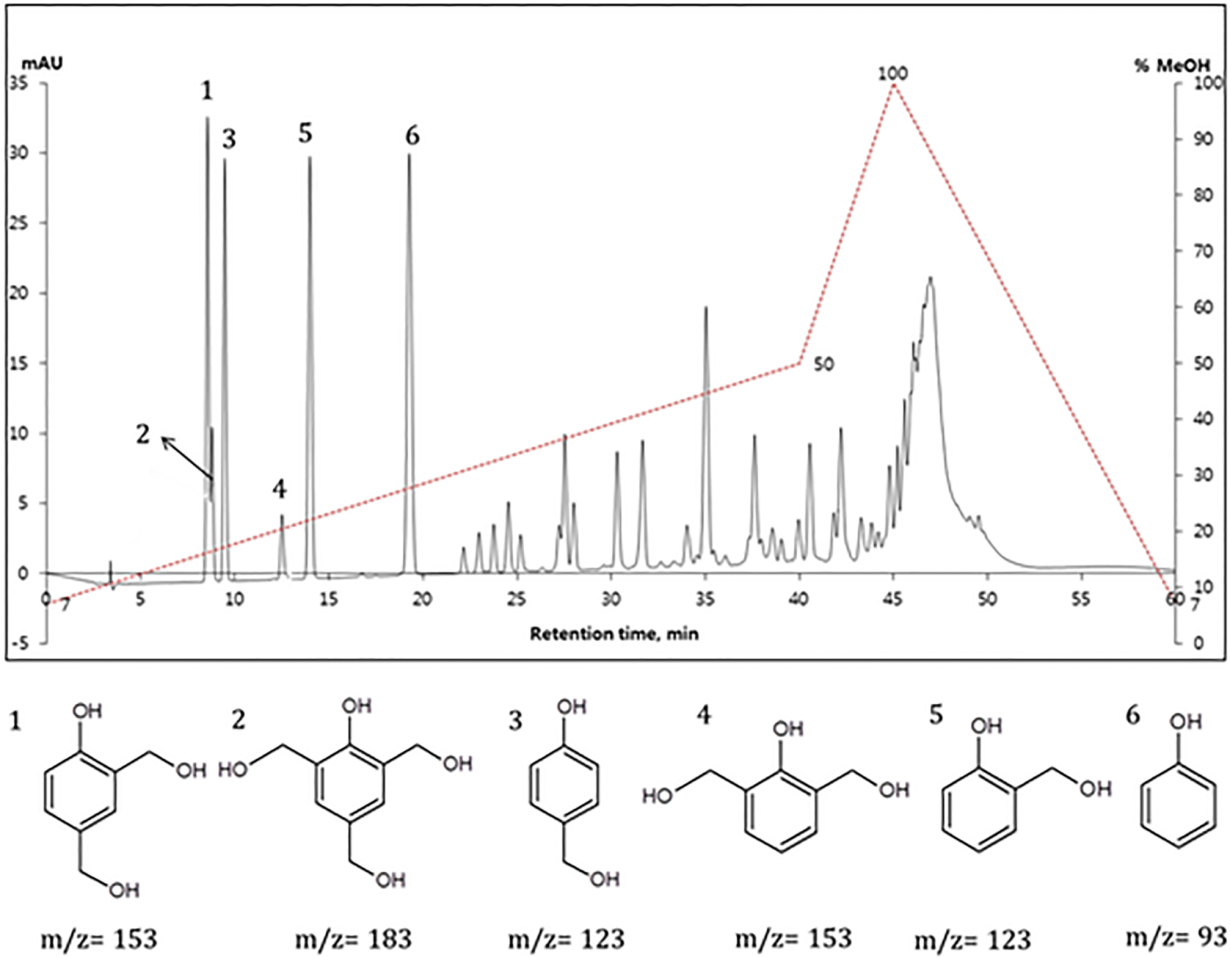
Chromatogram for a resol resin which was obtained in the presence of triethylamine as a catalyst.

The identified peaks for the HPLC/MS method were presented in Fig 1.

The analytical curve was established and used for the contents of phenol, 2-hydroxymethyl-phenol (2-HMP) and 4-hydroxymethylphenol (4-HMP), while concentrations of other components, i.e. 2,4-dihydroxymethylphenol (2,4-DHMP), 2,6-dihydroxymethylphenol (2,6-DHMP) and 2,4,6-trihydroxymethylphenol (2,4,6-THMP), were found with the internal standard method (4-ethylresorcinol).

The free formaldehyde content was found by the analytical curve method with the use of the derivatization reagent, i.e. 2,4-dinitrophenylhydrazine and the following conditions:

*Eluent*            - *water*, *methanol (40*:*60 v/v)*
*Flow rate*          - *1*.*0 ml/min*
*Measurement time*        - *30 min*
*Wavelength for UV/VIS detector* - *360 nm*

The types of analytical curves for quantitative analysis were selected on the basis of the (minimum) values of confidence intervals for the median. Linear relations were obtained in all cases for concentration of standard versus normalised peak area, over the measuring ranges adopted in the study.

## Result and discussion

### Experimental design

The following variability ranges were assumed for the synthesis parameters in the adopted experimental design type 3^2^ (Table 1).

**Table 1:** Variability ranges for parameters.

Parameter	Unit	Value
Synthesis temperature	°C	70–80
Amount of catalyst	mol/mol PhOH	0.03–0.06

The experimental design 3^2^ means that 2 factors are considered, each at 3 levels. These are usually referred to as low, intermediate and high levels. The software Statistica^*™*^ was used to generate a plan of 11 experiments, including 2 replicates in the central point, which covered the optimised parameters for the synthesis of resins [15]. The plan and the values of the response function were presented in Table 2.

**Table 2:** Experimental design.

Optimised parameters		Response functions					
TEA, [mol]	Temp., [°C]	PhOH, [%]	Σ (PhOH+HMP), [%]	CH_2_O, [%]	Viscosity, [mPas]	Non-volatiles, [%]	Gel. t._150_, [s]
0.06	80	12.41	34.71	0.04	147	62.2	190
0.06	70	13.62	52.17	0.26	49	60.56	216
0.045	75	13.76	47.75	0.13	61	59.3	217
0.06	75	13.72	45.55	0.09	73.5	60.1	201
0.03	75	14.81	52.41	0.33	45	57.7	245
0.03	70	16.13	62.14	0.66	38	56.8	284
0.045	80	11.97	35.22	0.08	110	62.3	200
0.045	70	14.63	55.35	0.38	47	59.2	250
0.045	75	13.98	48.3	0.1	65	59.6	228
0.045	75	13.82	47.89	0.12	64	59.9	222
0.03	80	11.68	36.89	0.19	76	62.02	208

Concentration of the following components in the final product: formaldehyde and total for phenol and its hydroxymethyl derivatives—∑(PhOH+HMP) i.e. PhOH + 2-HMP + 4-HMP + 2,4-DHMP + 2,6-DHMP + 2,4,6-THMP, were selected as key response functions.

**Fig 2:**
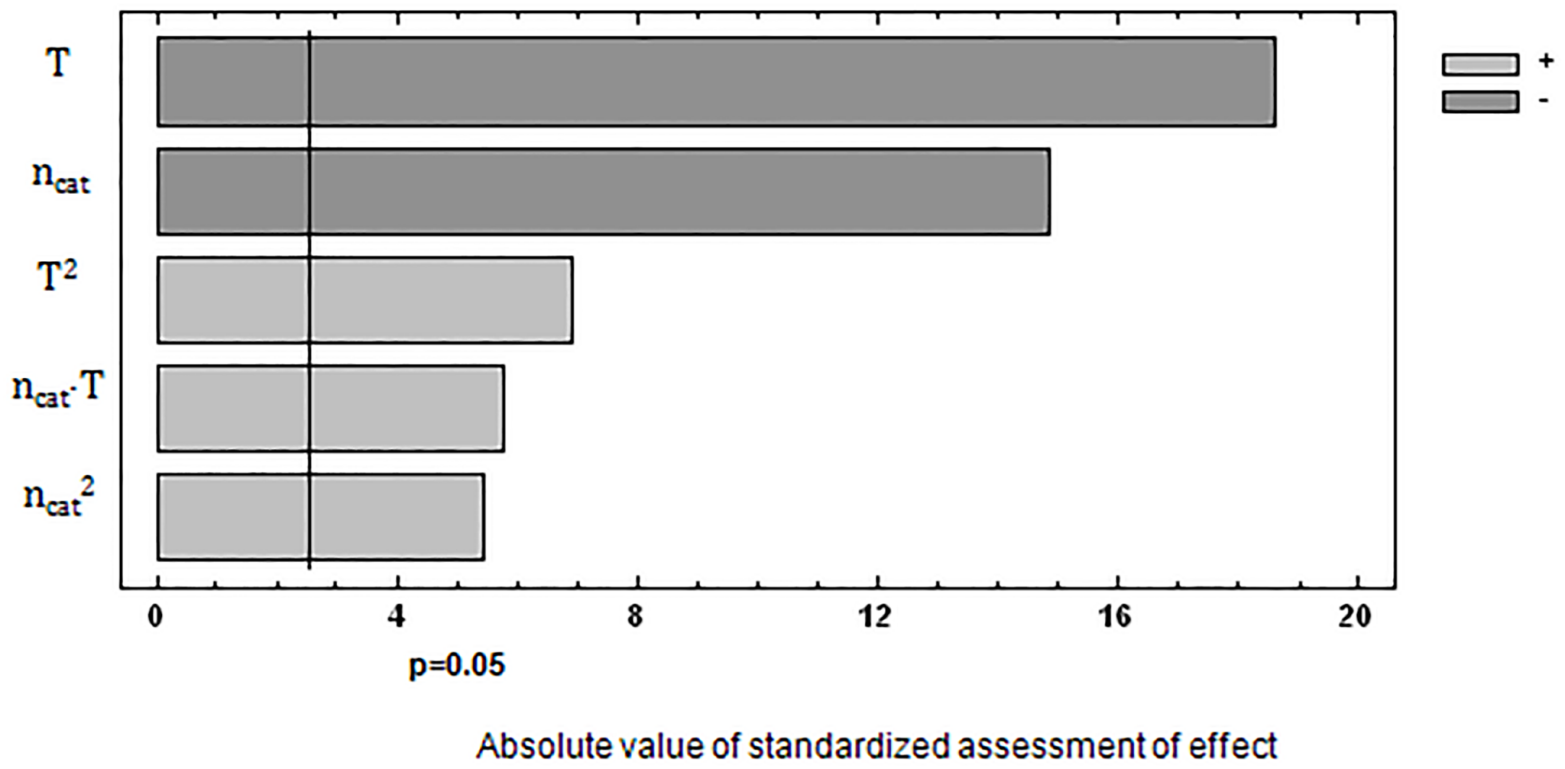
Significance of the influence of examined factors on concentration of CH_2_O.

**Fig 3:**
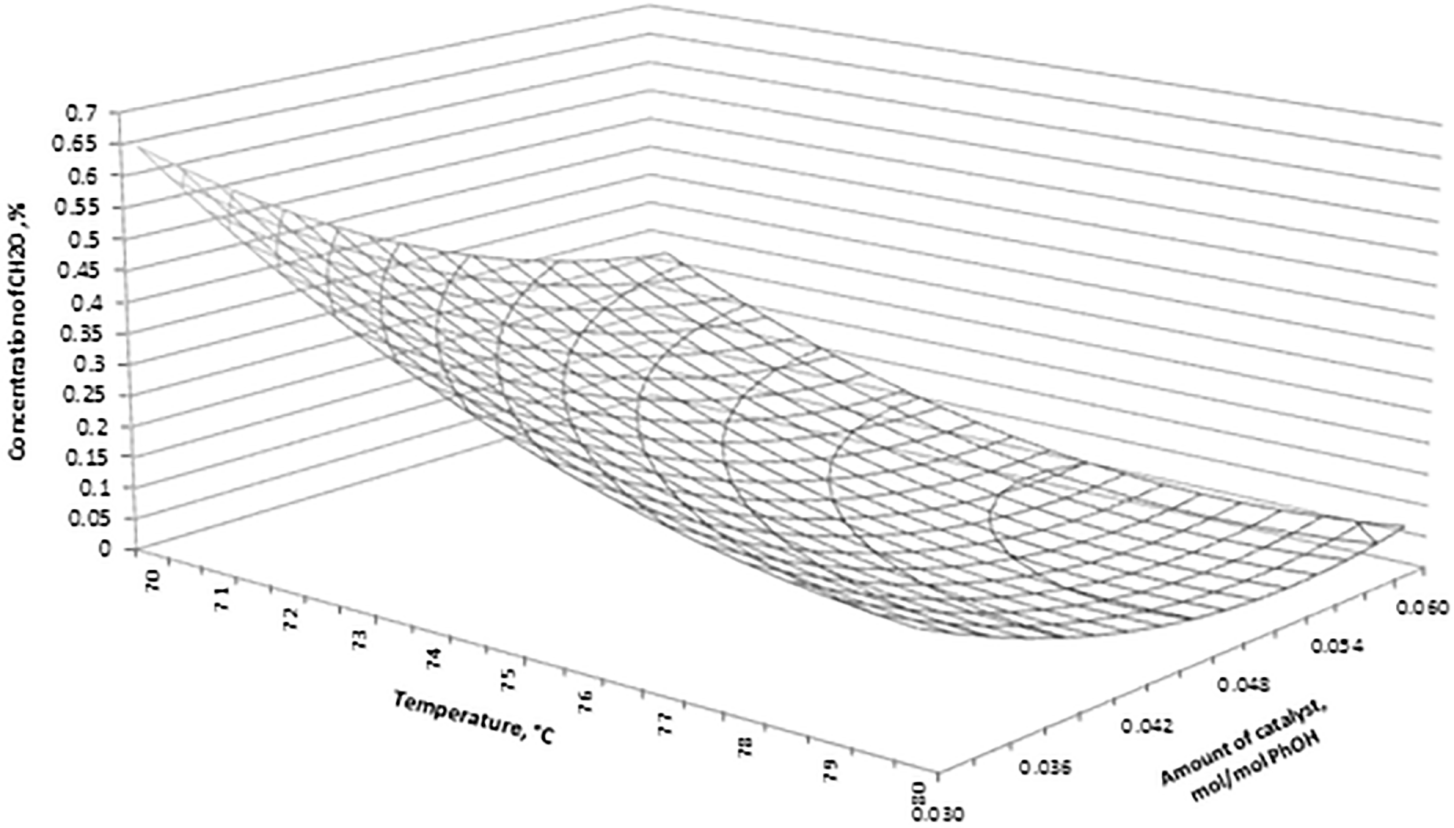
Concentration of CH_2_O versus amount of catalyst and synthesis temperature.

**Fig 4:**
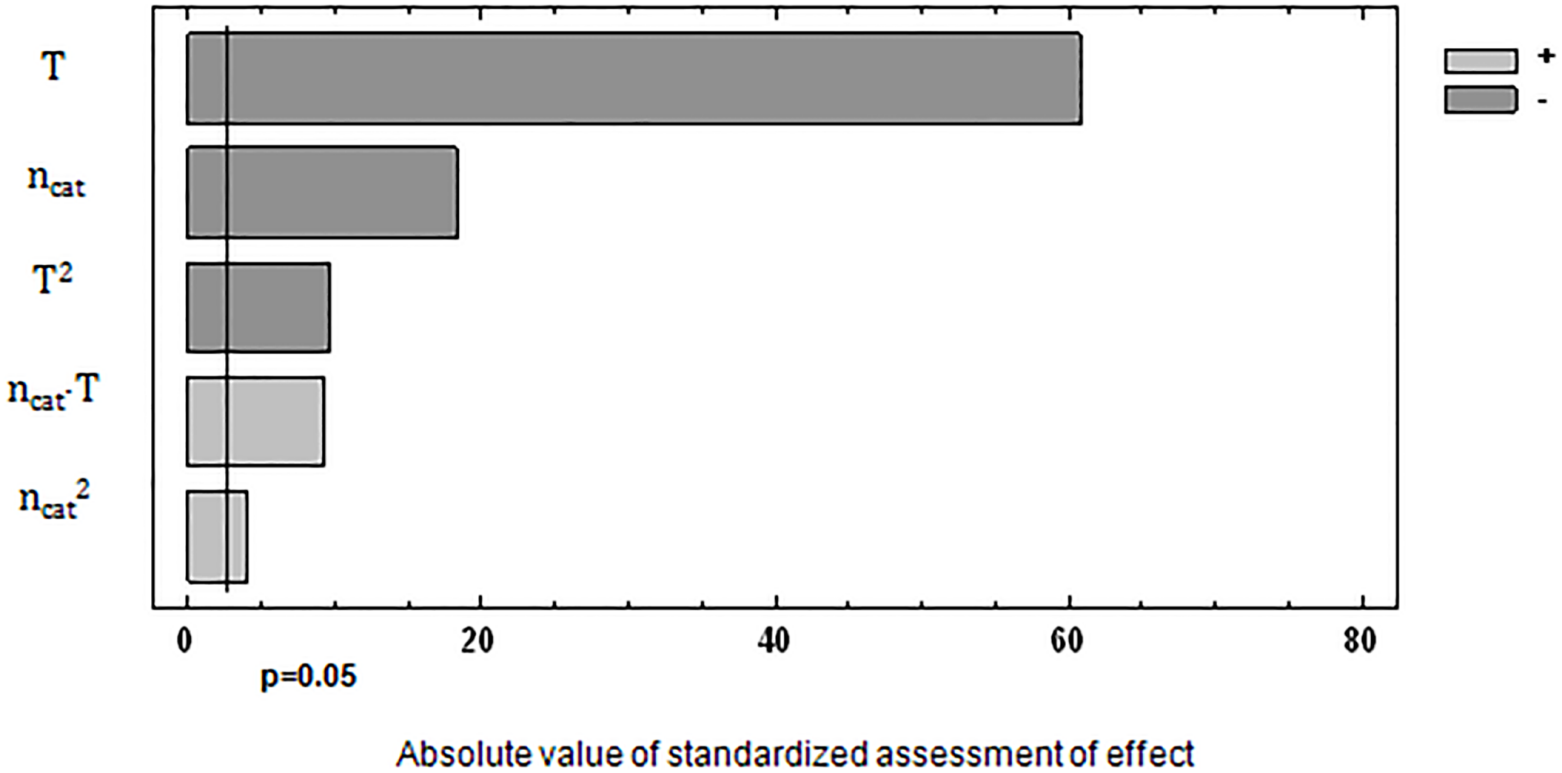
Significance of the influence of examined factors on concentration of Σ(PhOH+HMP).

**Fig 5:**
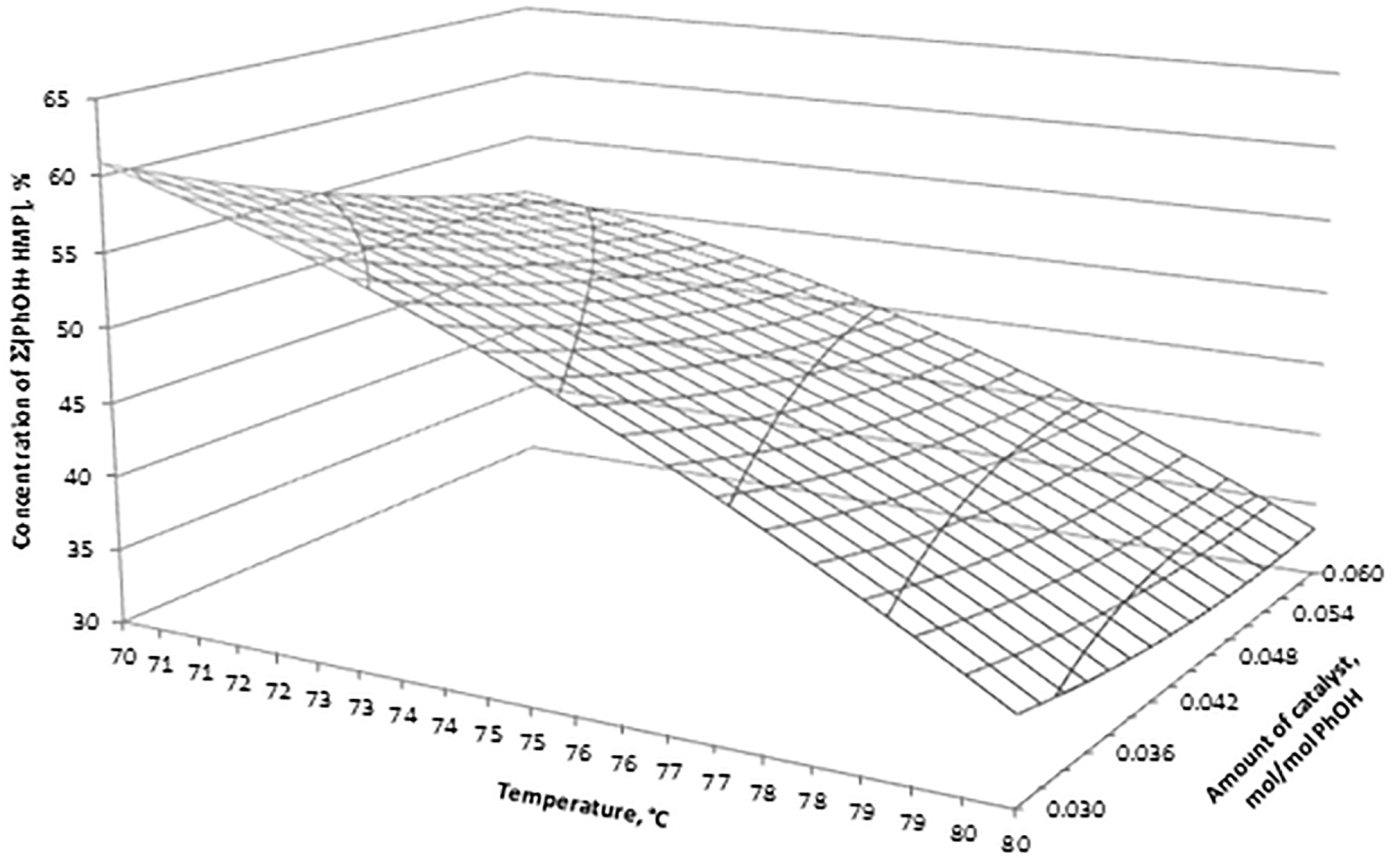
Concentration of Σ(PhOH+HMP) versus amount of catalyst and synthesis temperature.

The effect of significance of the studied process parameters on the response function was presented in the form of Pareto-Lorenz diagrams [16–18]. The bar length is proportional to the value of the analysed effect (calculated as a standardized value, i.e. divided by its standard error) in that type of diagram. The vertical line in that diagram stands for the initial border line for the significance of the effects of parameters on the response function value for the significance level α = 0.05. Then, the second order correlation functions were presented which contained essential factors (P < α) (Eqs 1 and 2) as well as coefficients of determination and standard errors of estimation. The significance of the factors and the charts for the obtained functional dependences was presented in Figs 2–5.

$${CH}_{2}O=27.73-101.07∙n_{cat}-0.64∙T+330.99∙n_{cat}^{2}+0.83∙n_{cat}∙T+0.004∙T^{2}$$ (1)

$${\text{R}}^{2}=99.30\%;\delta =0.02\%$$

The Table 3 presents the exact value of regression coefficients for Eq (1). The Table 4 compares the observed values with the fitted ones obtained from Eq (1).

**Table 3:** The exact value of regression coefficients for Eq 1.

*Coefficient*	*Estimate*
constant	27,7336
A: n_cat_	-101,067
B: T	-0,637342
AA	330,994
AB	0,833333
BB	0,00377895

**Table 4:** Estimation results for concentration of CH_2_O.

*Row*	*ObservedValue*	*FittedValue*
1	0,04	0,06
2	0,26	0,26
3	0,13	0,12
4	0,09	0,07
5	0,33	0,33
6	0,66	0,65
7	0,08	0,05
8	0,38	0,38
9	0,10	0,12
10	0,12	0,12
11	0,19	0,20

Fig 3 shows the interaction between the amount of catalyst and synthesis temperature on the concentration of CH_2_O. Optimum synthesis conditions and the predicted value of CH_2_O content resulting from Eq (1) are: n_cat_ = 0.054 mol/mol PhOH, T = 78,4°C, CH_2_O = 0.029%.

$$\sum (PhOH+HMP)=-271.49-2599.25∙n_{cat}+12.30∙T+4894.74∙n_{cat}^{2}+25.97∙n_{cat}∙T-0.10∙T^{2}$$ (2)

$${\text{R}}^{2}=99.8\%;\delta =0.42\%$$

Table 5 presents the exact value of regression coefficients for Eq (2). Table 6 compares the observed values with the fitted ones obtained from Eq (2).

**Table 5:** The exact value of regression coefficients for Eq 2.

*Coefficient*	*Estimate*
constant	-271,485
A: n_cat_	-2599,25
B:Temp	12,2989
AA	4894,74
AB	25,9667
BB	-0,103747

**Table 6:** Estimation results for concentration of Σ(PhOH+HMP).

*Row*	*ObservedValue*	*FittedValue*
1	34,71	34,75
2	52,17	51,81
3	47,75	47,94
4	45,55	45,87
5	52,41	52,21
6	62,14	62,04
7	35,22	34,87
8	55,35	55,82
9	48,30	47,94
10	47,89	47,94
11	36,89	37,20

Fig 5 shows the influence of the amount of catalyst and synthesis temperature on the concentration of Σ(PhOH+HMP). Optimum synthesis conditions and the predicted value of Σ(PhOH+HMP) content resulting from Eq (2) are: n_cat_ = 0.053 mol/mol PhOH, T = 80.0°C, Σ(PhOH+HMP) = 34.53%.

The statistical analysis, as presented above, shows that the determined contents of CH_2_O and Σ(PhOH+HMP), described by quadratic equations, are dependent on all the investigated factors over the studied range of variability for the synthesis parameters. All the coefficients in the correlation equations are statistically significant since their p-values are lower than the assumed significance level α = 0.05. The calculated values of coefficient determination in each case take on the values R^2^ > 99%.

The lowest content for formaldehyde, phenol and total phenol and its hydroxymethyl derivatives, i.e. CH_2_O = 0.04%, PhOH = 12.41% and Σ(PhOH+HMP) = 34.71%, was obtained for the following synthesis conditions: n_cat_ = 0.06 mol/mol PhOH and T = 80°C. The resin obtained under such conditions offered a low formaldehyde content, yet its content of phenol and hydroxymethyl derivatives remained excessively high. It was not advisable to further increase the amount of triethylamine in order to reduce the content of phenol and its derivatives, since the nitrogen content would rise to the level > 0.5%. Having in mind those limitations and the plan to add a polyamine co-catalyst to the synthesis processes, less severe synthesis conditions were assumed, i.e. TEA—0.03 mol/mol PhOH, temperature—75°C. The same conditions were also used for comparative syntheses which involved ammonia and selected polyamine catalysts and co-catalysts. Table 7 provides the process conditions as well as the compositions and physico-chemical properties of the obtained resins.

**Table 7:** Chemical composition and physico-chemical properties of resin (PhOH:CH_2_O:cat:co-cat = 1:1.15:0.03: 0.005, temp. 75°C).

Item	Catalyst [0.03 mol/mol PhOH]	Co-catalyst [0.005 mol/mol PhOH]	Time, [h]	Composition and physico-chemical properties					
				PhOH [%]	Σ(PhOH+HMP), [%]	CH_2_O [%]	Viscosity [mPas]	Non-volatiles [%]	Gel. t._150_ [s]
1.	TEA	-	5	14.81	52.41	0.33	45	57.70	245
2.	NH_3_	-	5	21.73	46.27	5.13	32	54.23	420
3.	DETA	-	2	7.97	10.03	2.50	> 10000	70.2	85
4.	TETA	-	1.75	7.09	9.52	2.67	> 10000	71.4	82
5.	TEA	DETA	5	8.55	24.9	0.07	475	63.02	147
6.	TEA	TETA	5	9.66	27.23	0.05	495	64.61	205

The results as presented in the table, show that the use of TEA makes it possible to produce a low-formaldehyde resin, however with high contents of phenol and its derivatives. The use of ammonia yielded a resin with a too low condensation level and very high content of objectionable components. In case polyamine catalysts were employed, the obtained resins offered very low content of phenol and hydroxymethylphenols, however the concentration of unreacted formaldehyde was much higher than 2.0%. Furthermore, the obtained resins failed to satisfy the application requirements, predominantly because of their high viscosity figures.

Admixture of a polyamine co-catalyst in the synthesis of resol resins made it possible to obtain the products with very low contents of formaldehyde, phenol and its hydroxymethyl derivatives, with good performance properties maintained at the same time.

### Kinetics and rate constant

The reaction of phenol with formaldehyde under basic conditions is a second-order reaction [19–24]. The rate of this reaction is described by the quadratic equation:

v=k∙[F]∙[P] (3)

where:

[F]—formaldehyde concentration, mol/dm^3^

[P]—phenol concentration, mol/dm^3^

k—reaction rate constant, dm^3^·mol^-1^·h^-1^

In order to normalize and simplify the experimental data appearing in Eq (3), the concentration of free formaldehyde is expressed as a function of the phenol concentration (Eq 4):

[F]=f([P]) (4)

After that assumption, Eq (3) can be presented as:

v=k∙f([P])∙[P] (5)

The differential form of this equation is as follows:

$$\frac{d[P]}{dt}=-k∙f(\left[P\right])∙[P]$$ (6)

and after separation of variables:

$$-\frac{d[P]}{\left[P]∙f(\left[P\right]\right)}=k∙dt$$ (7)

This equation can be bilaterally integrated within the limits of experimental data:

$$\int_{P_{0}}^{P_{i}}\frac{-d[P]}{\left[P]∙f([P]\right)}=k∙t_{i}$$ (8)

The subscript "0" means the initial condition (t = 0), while the subscript "i" means the conditions reached after the time t_i_.

When the integral on the left-hand side of the equation is denoted as I(P_i_), Eq (8) can be put down as:

$$I(P_{i})=k∙t_{i}$$ (9)

The slope of that linear equation makes the reaction-rate constant k, [dm^3^·mol^-1^·h^-1^].

Kinetic studies were carried out and the reaction-rate constants were determined for the syntheses in which conventional catalysts such as TEA and NH_3_ were used, and for the syntheses in which DETA and TETA as well as their mixtures with TEA were involved as catalysts.

The reaction mixture was sampled during the kinetic study at specific time intervals. Each sample was immediately placed in the freezing mixture, at approx. -30°C, thereby the chemical reactions were effectively slowed down until the samples could be analyzed.

The changes of the free formaldehyde concentration expressed as a function of the changes of the free phenol concentration may be expressed as follows:

1. NH_3_ [F] = 1/(-0.0687+1.3724/[P]); **R**^**2**^ **= 98.70%**
2. TEA [*F*] = −7.2128+5.7441·[*P*]^0.5^; **R**^**2**^ **= 99.62%**
3. DETA [*F*] = −2.4636+3.6606·[*P*]^0.5^; **R**^**2**^ **= 98.85%**
4. TETA [*F*] = 0.4454+1.1036·[*P*]; **R**^**2**^ **= 98.61%**
5. TEA+DETA [*F*] = −1.5427+1.4454·[*P*]; **R**^**2**^ **= 99.37%**
6. TEA+TETA [*F*] = −1.4721+1.4634·[*P*]; **R**^**2**^ **= 99.22%**

After substitution of the above relations into Eq (8) and after integration, the following equations were obtained:

1. NH_3_
  ∫P0Pi-1[P]-0.0687+1.3724[P]dP=1.3724[P]+0.06872∙ln[P]|P0Pi
2. TEA
  ∫P0Pi-1-7.2128∙[P]+5.7441∙[P]1.5dP=0.4159∙ln[P]-0.2773∙ln([P]1.5-1.2557∙[P])|P0Pi
3. DETA
  ∫P0Pi-1-2.4636∙[P]+3.6606∙[P]1.5dP=1.2178∙ln[P]-0.8118∙ln([P]1.5-0.673∙[P])|P0Pi
4. TETA
  ∫P0Pi-10.4454∙[P]+1.1036∙[P]2dP=2.2454∙ln([P]+0.4036)-2.2454∙ln[P]|P0Pi
5. TEA+DETA
  ∫P0Pi-1-1.5427∙[P]+1.4454∙[P]2dP=0.6482∙ln[P]-0.6482∙ln([P]-1.0673)|P0Pi
6. TEA+TETA
  ∫P0Pi-1-1.4721∙[P]+1.4634∙[P]2dP=0.6793∙ln[P]-0.6793∙ln([P]-1.006)|P0Pi

**Fig 6:**
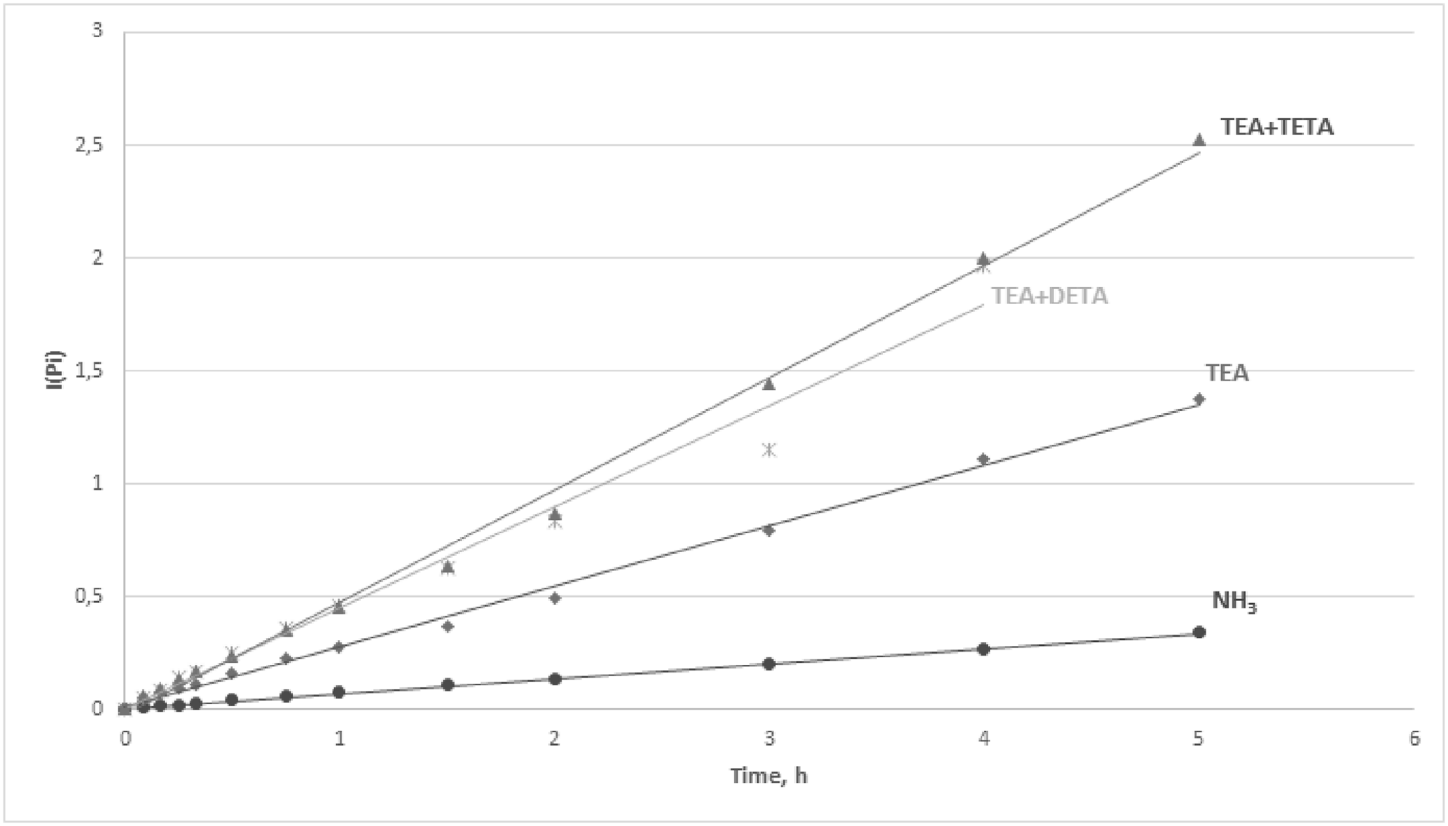
Values of integrals vs. synthesis time.

**Fig 7:**
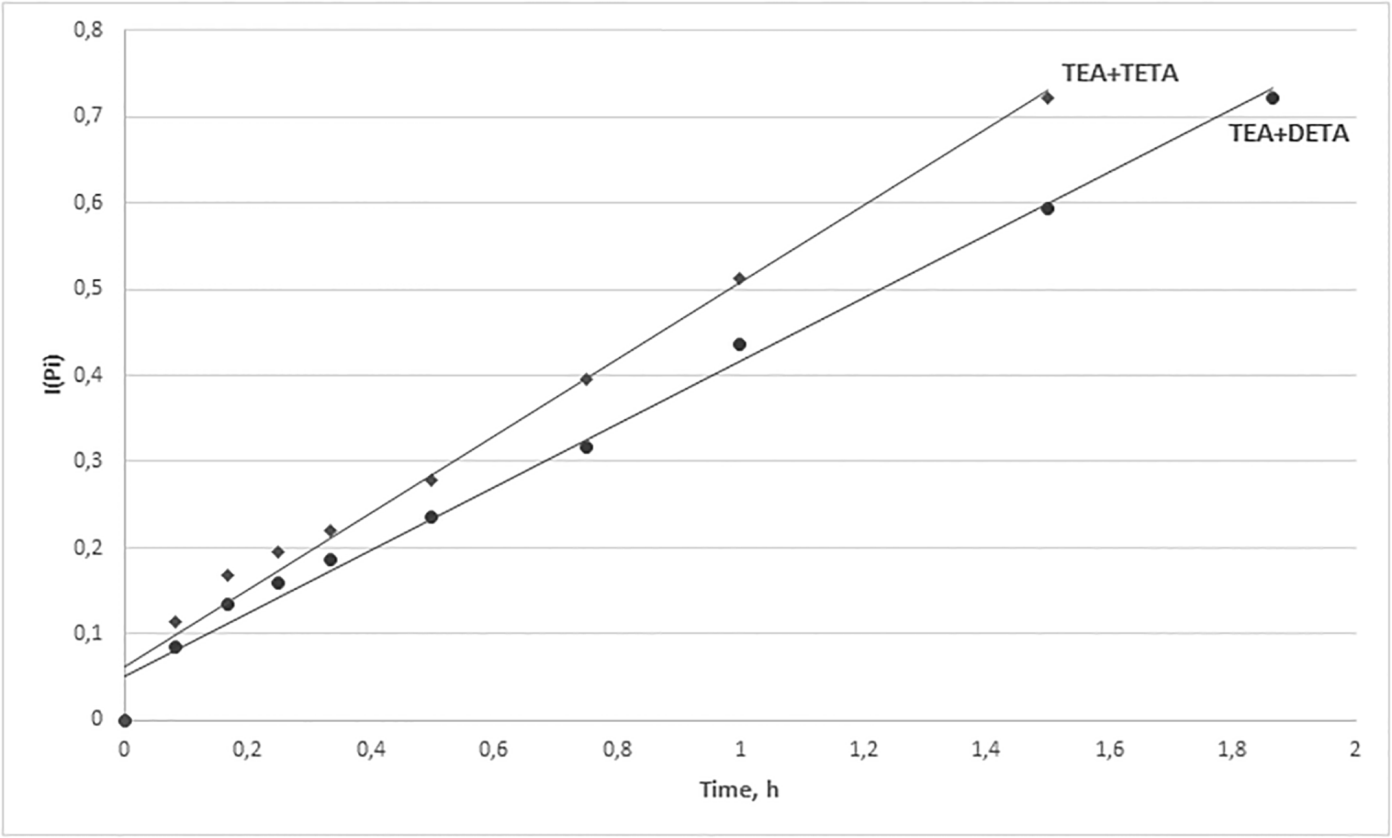
Values of integrals vs. synthesis time.

After calculating the values of the integrals in the range of P_0_ to P_i_, the obtained values of integrals versus synthesis time were plotted and presented in Figs 6 and 7.

The obtained equations *I*(*P*_*i*_) = *k* · *t*_*i*_ and the rate constants for the reactions in the presence of selected catalysts and catalytic systems were presented in Table 8.

**Table 8:** Equations, coefficients of determination and reaction rate constants values.

Item	Catalyst [0.03 mol/mol PhOH]	Co-catalyst [0.005 mol/mol PhOH]	Equations; R^2^	k, dm^3^·mol^-1^·h^-1^
1.	NH_3_	-	y = 0.0634x + 0.0090; R^2^ = 99.85	0.06
2.	TEA	-	y = 0.2678x + 0.0125; R^2^ = 99.62	0.27
3.	DETA	-	y = 0.3640x + 0.0523; R^2^ = 99.12	0.36
4.	TETA	-	y = 0.4455x + 0.0626; R^2^ = 98.51	0.45
5.	TEA	DETA	y = 0.4525x + 0.0012; R^2^ = 98.83	0.45
6.	TEA	TETA	y = 0.4971x − 0.0194; R^2^ = 99.61	0.50

The obtained equations were characterized by high coefficients of determination R^2^ > 98.0% and low values of standard estimation errors, which confirms that the proposed kinetic model for the reaction of phenol with formaldehyde in the presence of amine catalysts was valid. The rate constants were in the range 0.06–0.50 dm^3^·mol^-1^·h^-1^. The lowest values of rate constants were obtained for the synthesis with the use of conventional catalysts (NH_3_, TEA). Much higher values of rate constants were obtained when polyamine catalysts, i.e. DETA and TETA, were employed, but the level of polycondensation was increasing rapidly and made it impossible to carry out the synthesis over 5 hours as initially assumed. The highest values of rate constants, ≥ 0.45 dm^3^·mol^-1^·h^-1^, were available when the catalytic systems TEA+DETA/TETA were employed and the final products were characterized by the required degree of condensation.

### Conclusions

The research programme demonstrated that the type of the catalytic system had an essential effect on the composition and physico-chemical properties of the resins. The findings presented in this report showed that the use of triethylamine in the synthesis of resins made it possible to reduce the formaldehyde content down to the required level ≤ 0.5%. However, the content of phenol and hydroxymethylphenols remained excessively high. It was not advisable to increase the amount of triethylamine, since higher nitrogen contents in resins would adversely impact their performance properties. On the other hand, the use of polyamine catalysts yielded the products with low content of phenol and its hydroxymethyl derivatives, but with excessively high formaldehyde content. The syntheses which were catalysed by triethylamine and a polyamine as a co-catalyst produced resins with low content of both formaldehyde and phenol + hydroxymethylphenols; the physico-chemical properties and performance properties of those materials were as required at the same time. In case of adding diethylenetriamine, the contents of phenol and its hydroxymethyl derivatives could be reduced by 52.49% in relation to the resin obtained with the use of triethylamine and by 46.19% in relation to the resin obtained with the use of ammonia. The formaldehyde content were reduced by 78.79% and 98.64%, respectively. When triethylenetetramine was added as a co-catalyst, the content of phenol and its derivatives was reduced by 48.04% versus triethylamine-catalysed resin and by 41.15% versus ammonia-catalysed material. The reductions in formaldehyde content reached 84.85% and 99.03%, respectively.

The investigation proved that the unprecedented use of polyamine co-catalysts made it possible to produce resins with low content of formaldehyde, phenol and hydroxymethylphenols, which offer desirable physico-chemical properties and meet the application properties as required. That gives some prospects for commercialisation of the new synthesis method. The resins produced with the use of a polyamine catalyst, when employed in laminated materials, would offer measurable commercial profits and would bring environmental advantages as well.

A simplified method of calculation of the rate constant was adopted for the reaction of phenol with formaldehyde in the presence of amine catalysts. This method resulted from the complicated nature of the subsequent and parallel reactions and from the difficulties in analyzing fully the final product composition. The assumed calculation method for the reaction rate constants gave a very good approximation to the standard model.
